# Evaluation of surgically assisted rapid maxillary expansion with piezosurgery versus oscillating saw and chisel osteotomy - a randomized prospective trial

**DOI:** 10.1186/1745-6215-14-49

**Published:** 2013-02-17

**Authors:** Majeed Rana, Nils-Claudius Gellrich, Madiha Rana, Jozsef Piffkó, Wolfgang Kater

**Affiliations:** 1 Department of Cranio-Maxillofacial Surgery, Hannover Medical School, Carl-Neuberg-Str. 1, Hannover, 30625, Germany; 2 Department of Oral and Maxillofacial Surgery, University of Muenster, Muenster, Germany; 3 Department of Oral and Maxillofacial Surgery, Hochtaunus University Teaching Hospital, Urselerstraße 33, Bad Homburg, 61348, Germany

**Keywords:** Maxillary expansion, Piezosurgery, Saw, Neurologic, Pain

## Abstract

**Background:**

Ultrasonic bone-cutting surgery has been introduced as a feasible alternative to the conventional sharp instruments used in craniomaxillofacial surgery because of its precision and safety. The piezosurgery medical device allows the efficient cutting of mineralized tissues with minimal trauma to soft tissues. Piezoelectric osteotome has found its role in surgically assisted rapid maxillary expansion (SARME), a procedure well established to correct transverse maxillary discrepancies. The advantages include minimal risk to critical anatomic structures. The purpose of this clinical comparative study (CIS 2007-237-M) was to present the advantages of the piezoelectric cut as a minimally invasive device in surgically assisted, rapid maxillary expansion by protecting the maxillary sinus mucosal lining.

**Methods:**

Thirty patients (18 females and 12 males) at the age of 18 to 54 underwent a surgically assisted palatal expansion of the maxilla with a combined orthodontic and surgical approach. The patients were randomly divided into two separate treatment groups. While Group 1 received conventional surgery using an oscillating saw, Group 2 was treated with piezosurgery. The following parameters were examined: blood pressure, blood values, required medication, bleeding level in the maxillary sinus, duration of inpatient stay, duration of surgery and height of body temperature.

**Results:**

The results displayed no statistically significant differences between the two groups regarding laboratory blood values and inpatient stay. The duration of surgery revealed a significant discrepancy. Deploying piezosurgery took the surgeon an average of 10 minutes longer than working with a conventional-saw technique. However, the observation of the bleeding level in the paranasal sinus presented a major and statistically significant advantage of piezosurgery: on average the bleeding level was one category above the one of the remaining patients.

**Conclusion:**

This method of piezoelectric surgery with all its advantages is going to replace many conventional operating procedures in oral and maxillofacial surgery.

**Trial registration:**

CIS 2007-237-M

## Background

Transverse expansion of the maxilla was first done in 1860 by means of an orthodontic appliance. In the following decennia, the orthodontic treatment evolved. The theory of distraction was first published in 1905 by Codivilla [1]. The combined surgical and orthodontic treatment for maxillary expansion was introduced in 1938 for skeletally matured patients. The first successful use of distraction on the femur of a significant group of patients was published in 1990 [2]. In 1999, the first bone-borne distractor was introduced [3].

Once skeletal maturity has been reached, orthodontic treatment alone cannot provide a stable widening of the constricted maxilla in cases of deficiencies of more than 5 mm. In general, an orthodontist can camouflage transverse discrepancies less than 5 mm with orthopedic forces alone [4]. Tooth extractions for alignment of dental arches are often unnecessary [5]. As mentioned before, surgically assisted rapid maxillary expansion (SARME) is a form of distraction that was applied before its biological healing principles were known [5]. Physicians have to decide between two methods of expansion: SME (slow maxillary expansion) and RME (rapid maxillary expansion). Applying SME, the maxilla is broadened by 0.5 to 1 mm per week; meanwhile, using RME demands an expansion of 0.6 to 0.8 mm three times a day. Both methods have advantages and disadvantages [6]. The surgically assisted rapid maxillary expansion is a method which, using combined orthodontic-oral surgical treatment, leads to a distinctive extension of the midline palatal suture. Thereby, it is possible to avoid extractions, widen the nasal floor and support the change from oral to nasal breathing [7].

SARME is considered a procedure with little risk of serious complications; however, several complications are mentioned in the literature, varying from life threatening epistaxis to a cerebrovascular accident, skull base fracture with reversible oculomotor nerve pareses and orbital compartment syndrome [8-10]. Less serious complications reported are postoperative hemorrhage, pain, sinusitis, palatal tissue irritation/ulceration, asymmetrical expansion, nasal septum deviation, periodontal problems and relapse [11].

In 1976 Bell and Epker, as well as Neubert in 1989, described the surgically assisted maxillary expansion, all using an oscillating saw that injured the mucous membrane of the maxillary sinus [12]. Unfortunately, only a little information exists on how to preserve this mucous sinus membrane during the intervention. However, in 2001 Vercelotti described a new technique in osseous surgery which overcame the limits of traditional instrumentation in oral bone surgery by modifying and improving conventional ultrasound technology. Therefore, Vercelotti is known as the inventor of piezosurgery [13,14], a technique that allows the soft tissue to rest and a tendency for less bleeding [15-17]. It transmits a special modular ultrasonic vibration frequency on the scalpel. Not only is this technique clinically effective, but histological and histomorphometric evidence of wound healing and bone formation in experimental animal models has shown that tissue response is more favorable in piezosurgery than it is in conventional bone-cutting techniques, such as with diamond or carbide rotary instruments [18]. Voltage applied to a polarized piezoceramic causes it to expand in the direction of and contract perpendicular to polarity. A frequency of 25 to 29 kHz is used because the micromovements that are created at this frequency (ranging between 60 and 210 μm) cut only mineralized tissue; neurovascular tissue and other soft tissue is cut at frequencies higher than 50 kHz [19-22].

Piezoelectric devices are an innovative ultrasonic technique for safe and effective osteotomy or osteoplasty compared with traditional hard and soft tissue methods that use rotating instruments because of the absence of macrovibrations, ease of use and control, and safer cutting, particularly in complex anatomical areas. Its physical and mechanical properties have several clinical advantages: precise cutting, sparing of vital neurovascular bundles and better visualization of the surgical field. Piezoelectric bone surgery seems to be more efficient in the first phases of bony healing; it induces an earlier increase in bone morphogenetic proteins, controls the inflammatory process better, and stimulates remodeling of bone as early as 56 days after treatment [23].

SARME is reported to be performed under either general or local anesthesia, but with differences in surgical technique. Pterygo-maxillary separation is not recommended by those who have performed SARME under local anesthesia, as it is performed blindly and can produce profuse bleeding from the descending palatine bundle that is not easily controlled without a maxillary down-fracture [24,25]. Separation of the pterygoid junction is thus particularly useful if greater posterior expansion is desired [3]. The use of ultrasonic vibrations for fracture of the pterygoid plates during orthognathic surgery has been recently reported by Ueki *et al*. [26]. In other words, the scope of the study is to answer the question of whether a surgically assisted rapid maxillary expansion using Piezosurgery® (Mectron, Carasco, Italy) without using saws and chisels is as effective as one applying conventional procedures.

## Methods

Approval for the study was obtained from the relevant ethics committee at the University of Muenster, Germany (CIS 2007-237-M). In addition, positive written consent was obtained from each subject who participated in the study.

### Patients

A total of 30 consecutive adult patients (12 males and 18 females) with an indication for surgically assisted maxillary expansion were prospectively and observer-blind enrolled. The division into two groups occurred randomly. To reduce bleeding in the maxillary sinus, patients of the first group were treated conventionally with an oscillating saw, while patients of the second group were treated with a piezoelectrical saw. Exclusion criteria were syndromes like cleft or craniofacial deformities. Patients with systemic disorders, cardiac diseases, diabetes mellitus, epilepsy, infectious diseases, coagulative disorders, pregnancy and patients receiving any regular drug therapy (for example, antiphlogistic), except oral contraceptives, were excluded from the study.

### Surgical technique

**Figure 1:**
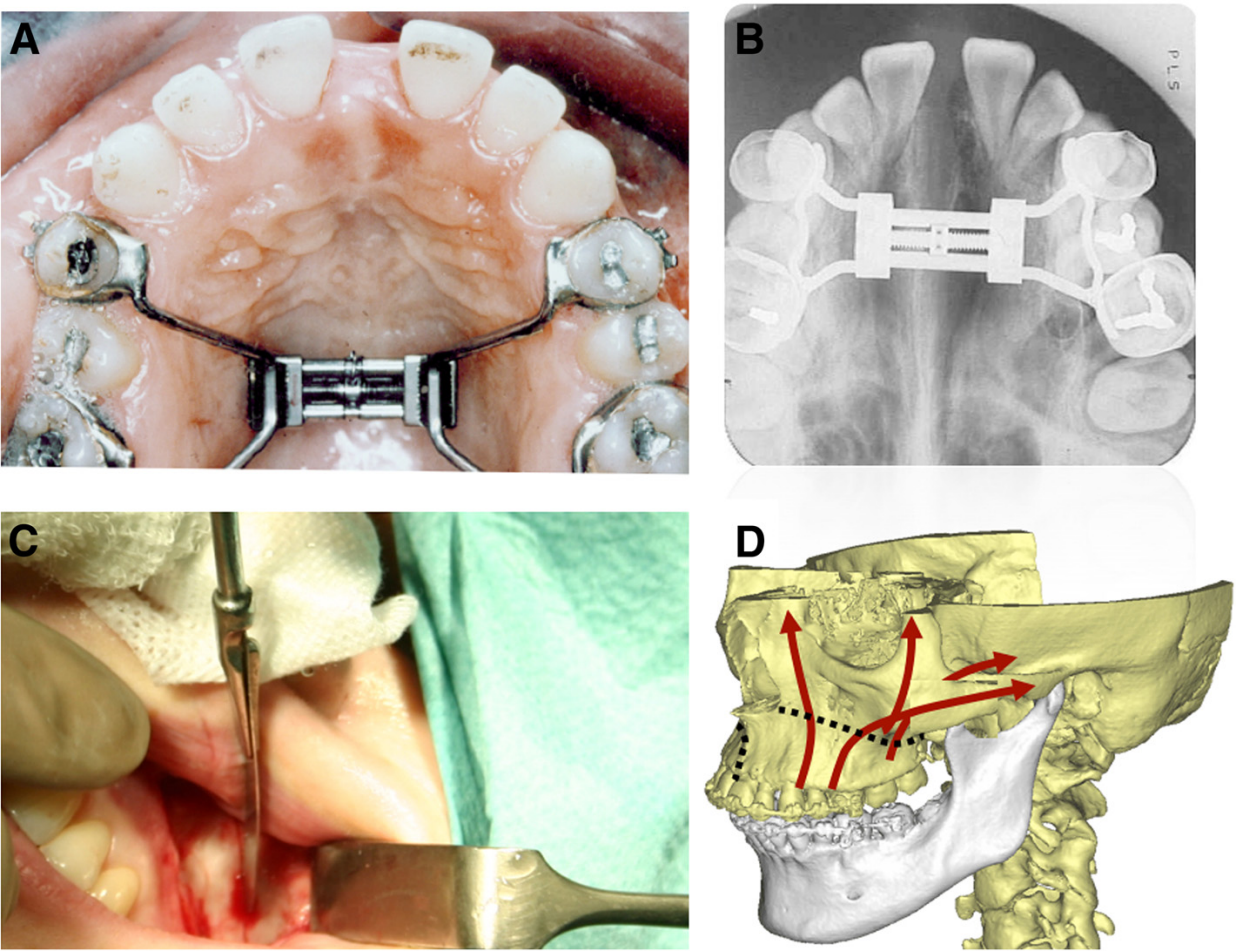
**(A) Demonstrates the distraction device connected with the postoperative visible diastema.** (**B**) Intraoral upper occlusion view shows the successful expansion of the maxilla postoperative. (**C**) Le-fort I osteotomy of the maxilla using a saw. (**D**) Schematic illustration demonstrating the osteotomy lines for surgical assisted rapid maxillary expansion.

Since patients were diagnosed with skeletal and dental malocclusion, all patients underwent preoperative orthodontics. All surgical interventions were conducted under general anesthesia with oral intubation and, in three cases, with nasal intubation to obtain better photographic documentation. The surgical procedure was standardized as Le-Fort 1 maxillary without the down-fracture technique as described by Epker [7]. The surgery was performed by one single surgeon. Drug therapy in both groups included 1,000 mg paracetamol (Perfalgan®) intravenously and 100 mg diclofenac (Voltaren®, Novartis, Munich, Germany) per day for two days from the second post-operative day as anti-inflammatory and analgesic therapy. Antibiotic prophylaxis consisted of Rocephin (Roche, Grenzach-Wyhlen, Germany) 2,000 mg per day for two days. Perioperatively, only a single dose of 50 mg steroids (Solu Decortin® (Merck Pharma GmbH , Darmstadt, Germany) was administered to every patient (Figure 1).

### Piezosurgical medical device

**Figure 2:**
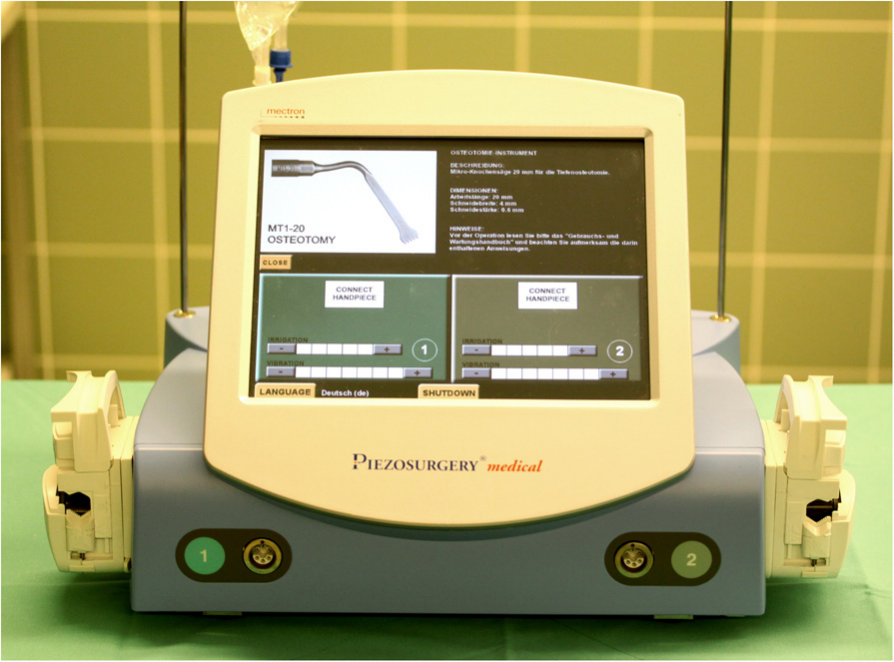
**Piezosurgery® medical, multipurpose device uses micrometric ultrasonic piezoelectric vibrations, variable in frequency and in cutting energy.** There is a therapeutic potential and benefit of the piezoelectric-assisted surgical saw in dentoalveolar surgery. Piezosurgery® enables more precise and nontraumatic cutting of bone in comparison to conventional methods (micrometic cut). The instrument vibrates with a modulated ultrasonic frequency. Because the vibration frequency of Piezosurgery® is optimal for mineralized tissue, it does not cut soft tissue. Therefore, an osteotomy with this device to remove a bony mass of the mandible prevents anatomic soft tissue injuries, such as to the dentoalveolar nerve (Figure 3).

**Figure 3:**
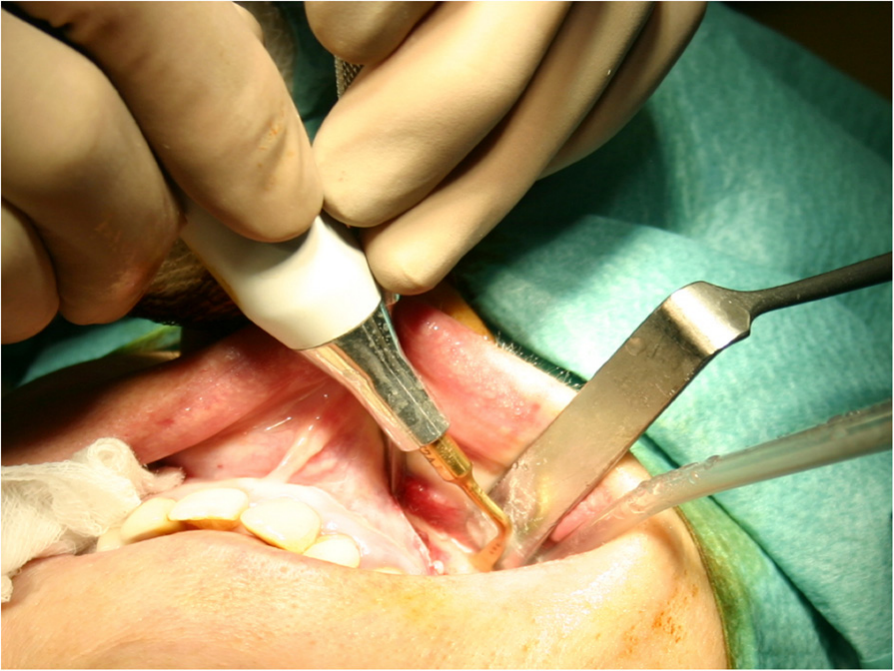
Le-fort I osteotomy using Piezosurgery® medical device with the OT 7A insert.

Piezosurgical medical device is a multipurpose device that uses micrometric ultrasonic piezoelectric vibrations, variable in frequency and in cutting energy. The device consists of a platform with a powerful piezoelectric hand piece and uses a functional frequency between 25 and 29 kHz with the possibility of digital modulation (boosted) up to 30 kHz. The device is also fitted with a cooling irrigation system with a 0 to 60 μL/min of variable sterile solution flow. Specific inserts and scalpels act in a linear vibration pattern, with the spatial range included between 60 and 210 μm, moved by ultrasonic power that exceeds 5 W, reaching also 16 W (Figures 2 and 3).

### Consort flow diagram

At the time of presentation, 48 patients were assessed for eligibility to be included in the study. Out of these, 20% of the patients (n = 10) were not included in the study as 12% of patients (n = 6) did not meet the inclusion criteria while 8% (n = 4) did not want to participate in the study. A total of 38 patients were randomly allocated in two groups with 19 patients allocated in each group for intervention. In the group treated by an oscillating saw 100% of the patients (n = 19) received the selected intervention. In the group operated on with the piezoelectric device 100% patients (n = 19) received the selected intervention. Among the 19 patients who were operated on using an oscillating saw, 15% (n = 3) were lost to follow-up as these patients came from far areas and could not travel due to economic or personal reasons. Of the 19 patients who were managed using the piezoelectric device, 5% (n = 1) were lost to follow-up. The 16 patients who received treatment using an oscillating saw in Group 1 were available for follow-up; 1 of them had their data lost during the data analysis procedure. So the total number of patients who were analyzed for an oscillating saw was 15.

**Figure 4:**
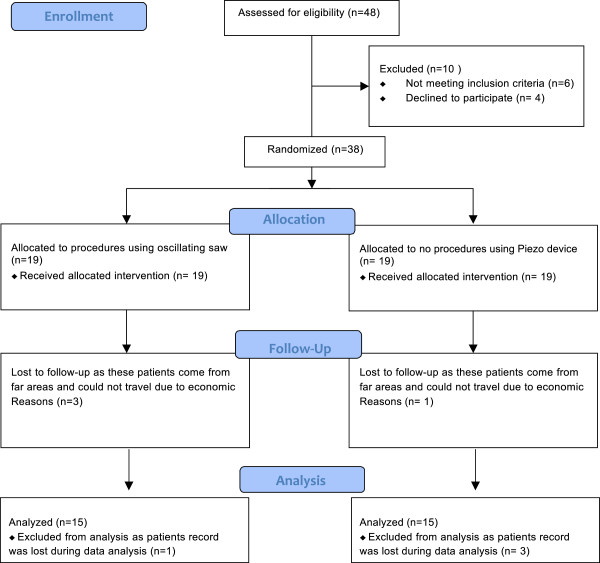
Demonstrates consort flow diagram.

Of the 18 patients who were managed using the Piezosurgery® device in Group 2 and were available for follow-up, 3 had their data lost during the data analysis procedure. So the total number of patients who were analyzed for piezosurgery was 15 (Figure 4).

### Randomization

Randomization was done using a computer-based software “EpiCalc2000” (Brixton-Health, London, United Kingdom). The software was used to generate serial numbers 1 to 100 randomly into two groups and those patients who fulfilled the inclusion criteria were allocated serial numbers according to date and sequence of admission to the hospital. The person responsible for conducting the measurements at the time of assessment of variables was blinded regarding the type of procedure that was conducted.

### Sample size

The sample size for the study was planned using the data of a pretest (n = 10). In this pretest, the alpha error level was set as 0.05 with 95% confidence level and a study power of 80%. The hematosinus of patients after definitive surgical assisted maxillary expansion of hard and soft tissue in orthgnathic patients was 11.9 + 5.8 mm in Group 1 and 5.7 ± 2.6 mm in Group 2. The calculated sample size was 15 cases in each group with an indication for surgically assisted maxillary expansion.

### Blood loss and hematosinus

Blood loss and hematosinus was measured using the paranasal sinus exposures preoperative and direct postoperative. A potential hematosinus was noticed by the measurement of the filling level of the maxillary sinus.

### Post-operative pain analysis

Pain analysis was performed using a visual analogue scale as described previously [23]. Briefly, pain was graded on a scale from 0 to 10, where a 0 denotes no pain and 10 maximum intensity of pain.

### Post-operative neurological score analysis

Neurological analysis was done for infraorbital nerve bilaterally as described previously [6] with some modifications. This method was basically created for nerve reconstructions. We used this neurological score to evaluate nerve dysfunction after orthognathic surgery. Briefly, the skin of the infraorbital region and the upper lip was checked using a cotton test for touch sensation, a pinprick test using a needle for sharp pain and a blunt instrument for pressure. In addition, a two-point discrimination test was performed on these regions. The results were graded on a score ranging from 0 to 13, where 13 was set as the worst neurological score. The scores were collected on the fourth day and at six months postoperatively.

### Patient satisfaction with surgical treatment

All patients were given a questionnaire before discharge from the hospital. Patients were questioned based on the subjective perception of the comfort and satisfactory concerning the post-operative cooling therapy. The data were graded in a scale ranging from 1 to 4, with 1 set as very satisfied and 4 not satisfied.

### Statistical analysis

All data are expressed as mean values ± 1 SEM (standard error of the mean). A one-way analysis of variance (ANOVA) with *post hoc* Bonferroni’s test for multiple comparisons of means was used for repeated measures. The Student’s *t*-test was applied for quantitative variables. A *P-*value *<*0.05 was considered significant. Statistical analysis was done with SPSS software for windows Version 14.0 (SPSS Inc., Chicago, IL, USA).

## Results

### Baseline characteristics

Thirty patients were analyzed in this clinical comparative study. Fifteen patients were assigned to Group 1 and underwent a conventional surgery using an oscillating saw. The remaining 15 patients belonged to Group 2 and were operated on with the piezosurgery device. The clinical and demographic characteristics of the patients from both groups are shown in Table 1. No significant differences were found in both groups regarding gender, age, blood values, pre- and postoperative body core temperature and blood pressure, and hospital stay duration. However, operation duration was significantly longer with the piezosurgery device compared to the oscillating saw.

**Table 1:** Baseline characteristics of patients

**Parameters (unit) ± SD**	**Oscillating saw**	**Piezo-device**	***P-*value**
	**Group 1 (n = 15)**	**Group 2 (n = 15)**	
Gender male – no. /total no. (%)	9/15 (60)	6/15 (40)	0.217
Age (years)	27.4 ± 9.4	29.8 ± 11.3	0.424
**Pre-operative**			
Haemoglobin (g/dl)	14.63 ± 1.41	13.79 ± 0.87	0.103
Haematocrit (%)	42.5 ± 3.8	40.2 ± 3.5	0.144
Body core temperature (°C)	36.2 ± 0.2	36.3 ± 0.3	0.271
Blood pressure (mmHg)	118.7 ± 9.9	123.3 ± 10.5	0.290
**Post-operative**			
Haemoglobin (g/dl)	13.75 ± 1.19	12.83 ± 0.87	0.058
Patients satisfaction	3.1 ± 0.3	1.9 ± 0.2	0.003

### Blood loss and hematosinus

Blood loss during surgery added up to a mean of 0.87 ± 0.68 g/dl for Group 1 and 0.95 ± 0.65 g/dl for Group 2. The blood loss during surgery with both operative techniques remained without statistical significance (*P* = 0.784).

**Figure 5:**
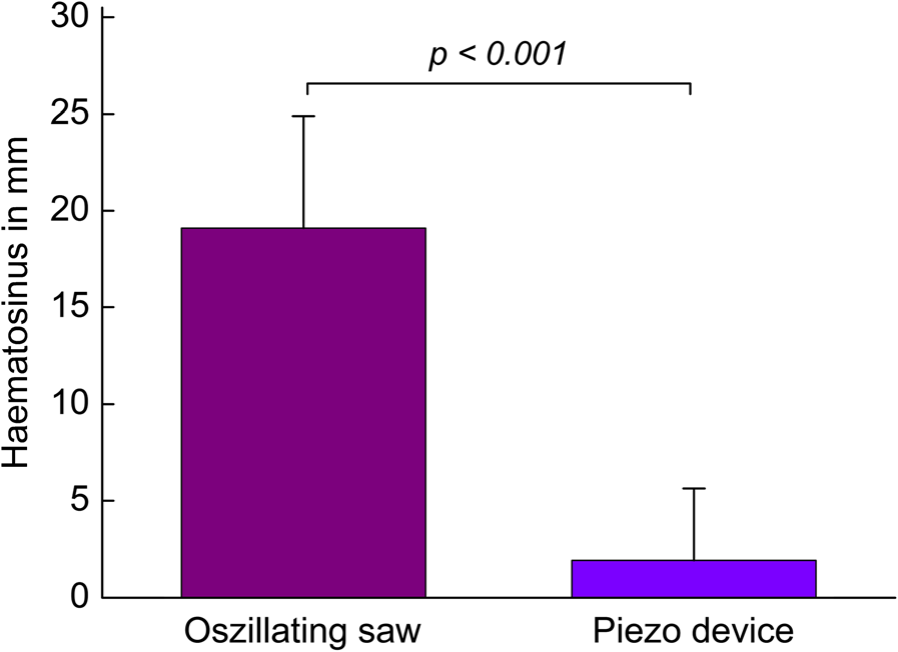
**Using the paranasal sinus exposures a potential hematosinus could be traced and the filling level of the maxillary sinus could be determined.** A significant difference between the two treatment methods (significance level *P* <0.001)*.*

Using the paranasal sinus exposures, a potential hematosinus could be traced and the filling level of the maxillary sinus could be determined. All patients in Group 1 displayed a hematosinus with mean filling heights of 19.1 ± 5.8 mm. As a contrast, among the Group 2 patients that received piezotherapy, only four patients exhibited a hematosinus with a mean filling level of 1.9 ± 3.7 mm. The Student’s *t*-test proves that there exists a significant difference between the two treatment methods (significance level *P* <0.001) (Figure 5).

### Postoperative pain

**Figure 6:**
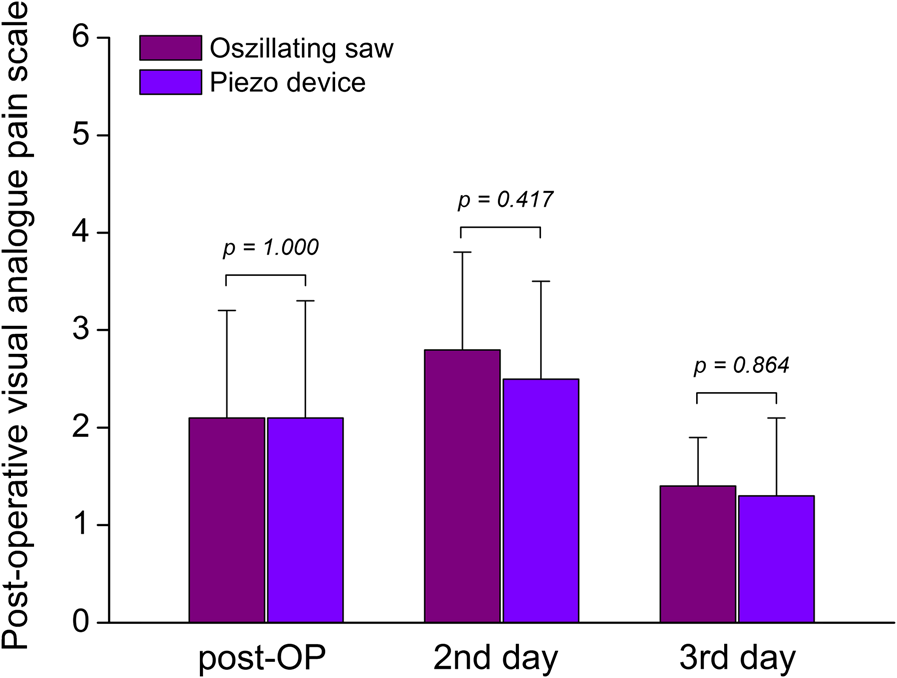
**Pain was calculated in terms of a visual analogue scale from subjective analysis ranging from 0 to 10.** A significant increase of pain was not found in the conventional group compared to the Piezosurgery® group during all examined post-operative days. No significant differences were observed between both groups.

Pain was calculated in terms of a visual analogue scale from subjective analysis ranging from 0 to 10. Post-operatively (Group 1: 2.1 ± 1.1, Group 2: 2.1 ± 1.2, *P* = 1.0), at the second postoperative day (Group 1: 2.8 ± 1, Group 2: 2.5 ± 1, *P* = 0.417) and the third postoperative day (Group 1: 1.4 ± 0.5, Group 2: 1.3 ± 0.8, *P* = 0.864) no significant differences were observed between both groups (Figure 6).

### Postoperative neurological score

**Figure 7:**
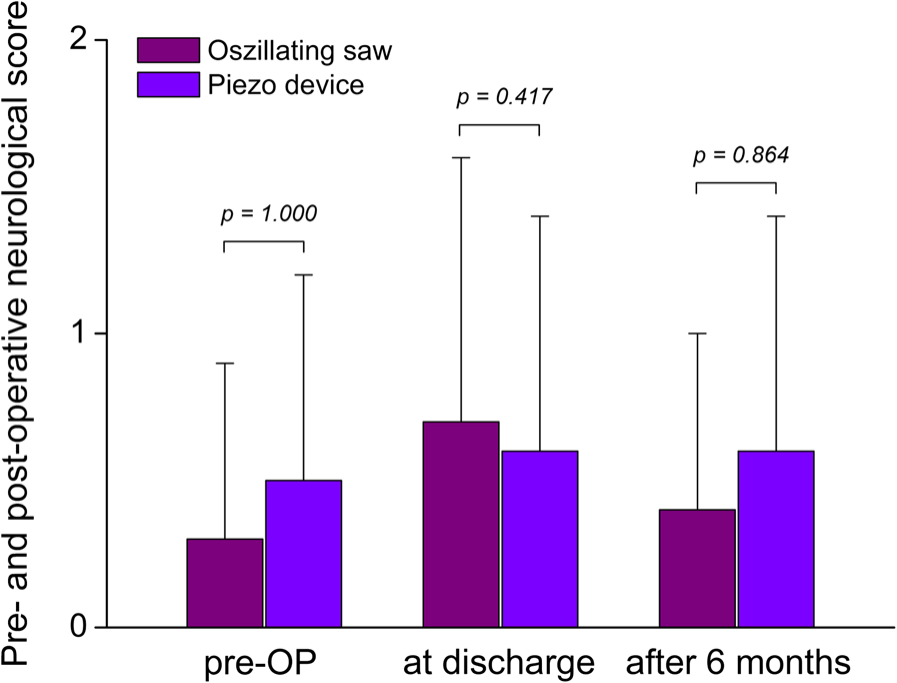
There were no statistically significant differences found between both groups concerning the neurological score pre-operatively and six months after surgery.

There were no statistically significant differences found between groups concerning the neurological score pre-operatively (Group 1: 0.3 ± 0.6, Group 2: 0.5 ± 0.7, *P* = 0.458), at discharge (Group 1: 0.7 ± 0.9, Group 2: 0.6 ± 0.8, *P* = 0.698) and six months after surgery (Group 1: 0.4 ± 0.6, Group 2: 0.6 ± 0.8, *P* = 0.337) (Figure 7).

### Post-operative soft tissue swelling

The swelling of the soft tissue was not very intense and disappeared within the first three post-operative days with the help of hilotherapy. The maximum degree of swelling was reached on the second post-operative day.

### Patient satisfaction

Regarding the patient’s satisfaction, which was assessed at the second day after surgery, a statistically significant difference between Piezosurgery® and the conventional saw could be detected (Group 1: 3.1 ± 0.3, Group 2: 1.9 ± 0.2, *P* = 0.003) Table 1.

## Discussion

The main scope of this clinical, comparative study was to evaluate whether a surgically assisted rapid maxillary expansion using piezotechnology is at least as effective and good as using a conventional oscillating saw.

In the course of the development of orthodontics and maxillofacial surgery different methods for maxillary expansion were tried. When having a closer look at the corresponding literature one sees that no exact indications of how to accomplish a maxillary expansion are defined. The success of a maxillary expansion has been known for centuries, but its efficacy for early treatment cannot yet be rated. As a rule, orthodontists urge an early start of therapy because at that time the growing activity of the bones can be utilized. Nevertheless, there are serious limits, such as orthognathic deseases beyond which an orthodontist should contact a maxillofacial surgeon. In reality, this happens rarely. A similar issue arises when orthodontists do not recommend a maxillary expansion because it requires surgery and, therefore, contains some risk, but instead prefer the extraction of healthy premolars.

The applied surgical method resembles, except for some smaller modifications, the method used by Bell and Epker [7,15-19].

The control of the laboratory blood values was conducted by the author himself, as well as the interventions applying piezosurgery. The conventional and piezoelectrical surgeries were accomplished by another surgeon. Therefore, a short learning curve was necessary to get used to the new cutting device. Since piezosurgery needs some time of familiarization, the first five piezosurgery patients were not included in this study.

Due to the exact incision and accurate osteotomy, no arterial vessels were injured and, consequently, no major bleeding occurred. Both patient groups lost approximately the same amount of blood, but since piezosurgery took an average of 10 minutes longer, for Group 2 the loss of blood per unit of time was less than for Group 1. For the author and his team the post-operative condition of the patients that were operated on with the piezo-saw was unambiguous. Some of them were physically more active after a much shorter period of time than Group 1 patients and could, therefore, leave the hospital earlier. However, some patients of Group 2 required a higher degree of pain medication and had to stay another day. A better method for patient selection would have been selecting according to age and gender. This way it would have been easier to distinguish between objective and subjective pain of the different age and gender groups. Due to the random assignment, Group 2 included more females and a larger number of young people. Presumably this is the reason why Group 2 displayed more pain and more circulatory problems. This assumption can be affirmed by the fact that within Group 1 the younger and female patients exhibited more pain and circulatory problems as well. Nevertheless, this study worked with people of all ages to guarantee that different anatomical bone structures with diverse bone qualities had to undergo osteotomy.

The spectrum of maxillofacial procedures has broadened significantly during the last centuries and the interventions have become more and more complex and invasive [27-29]. This is why it became and still is more important to develop operating techniques that are more precise and gentle [30,31]. Piezoelectric surgery could represent one of those techniques. The results of many piezoelectric osteotomies according to Vercelotti *et al*. [15-17,32,33], Borman *et al.*[34], Robiony *et al*. [35] and many more encourage the author to apply the piezoelectric osteotomy device for even more indications. It is histologically provable that in the long run wound healing is better with this procedure than after surgery with an oscillating saw [14]. Piezosurgery is not a method for fast surgeries, but it is suitable for sensitive and non-traumatic operation procedures.

Neither the laboratory values nor the duration of inpatient stay differed significantly between the two groups. Looking at the height of the blood level in the paranasal sinus piezosurgery showed a statistically significant difference. In conclusion, the question that was posed at the beginning of this paper can be given a positive answer. It is possible to conduct a surgically assisted rapid maxillary expansion with the help of an ultrasonic-saw, piezosurgery, which preserves the mucous membrane of the maxilla and is at least as effective and good as the conventional method. The high performance in terms of frequency and power of the piezosurgical device allow it to be used without the aid of any other osteotome, and with the same atraumatic effect on critical vascular structures. The very low amount of bleeding observed during surgery, lack of damage to the main vessels and reduction of postoperative consequences (hematomas, swelling) for patients were striking.

## Conclusion

It is possible to do a surgically assisted palatal expansion of the maxilla by using an ultrasonic bone-cutting device and thus protect the mucous membranes of the maxilla. This method of piezoelectric surgery with all its advantages is going to replace many conventional operating procedures in oral and maxillofacial surgeries.

### Ethical approval

Approval for the study was obtained from the relevant ethics committee at the University of Muenster, Germany (CIS 2007-237-M). In addition, positive written consent was obtained from each subject who participated in the study.

## Abbreviations

RME: Rapid maxillary expansion; SARME: Surgically assisted rapid maxillary expansion; SME: Slow maxillary expansion

## Competing interests

The authors declare that they have no competing interest.

## Authors’ contributions

MR, NCG, MAR, JP and WK conceived of the study and participated in its design and coordination. MR and WK made substantial contributions to conception and design of the manuscript as well as data acquisition. NCG and JP were involved in revising the manuscript. MR drafted the manuscript. MAR made the statistical analysis. All authors read and approved the final manuscript.
